# Current Understanding of the Formation and Adaptation of Metabolic Systems Based on Network Theory

**DOI:** 10.3390/metabo2030429

**Published:** 2012-07-12

**Authors:** Kazuhiro Takemoto

**Affiliations:** 1 Department of Bioscience and Bioinformatics, Kyushu Institute of Technology, Kawazu 680-4, Iizuka, Fukuoka 820-8502, Japan; Email: takemoto@bio.kyutech.ac.jp (K.T.); Tel.: +81-948-29-7822; Fax: +81-948-29-7801; 2 PRESTO, Japan Science and Technology Agency, Kawaguchi, Saitama 332-0012, Japan

**Keywords:** metabolic network, large-scale network analysis, evolution, environmental adaptation

## Abstract

Formation and adaptation of metabolic networks has been a long-standing question in biology. With recent developments in biotechnology and bioinformatics, the understanding of metabolism is progressively becoming clearer from a network perspective. This review introduces the comprehensive metabolic world that has been revealed by a wide range of data analyses and theoretical studies; in particular, it illustrates the role of evolutionary events, such as gene duplication and horizontal gene transfer, and environmental factors, such as nutrient availability and growth conditions, in evolution of the metabolic network. Furthermore, the mathematical models for the formation and adaptation of metabolic networks have also been described, according to the current understanding from a perspective of metabolic networks. These recent findings are helpful in not only understanding the formation of metabolic networks and their adaptation, but also metabolic engineering.

## 1. Introduction

Because metabolism is responsible for physiological functions and for maintaining life, it is an important topic not only in general biology but also in applied biological research fields such as biotechnology and medical science. In recent years, several new technologies and high-throughput methods have generated considerable genomic and metabolic data. The data regarding metabolic information are collected in several databases such as the Kyoto Encyclopedia of Genes and Genomes (KEGG) [1] and the Encyclopedia of Metabolic Pathways (MetaCyc) [2]; these databases are widely used and contain the metabolic pathways of many living organisms. Thus, the overall picture of the metabolic world has gradually become clearer.

How should we capture this metabolic world? When addressing such a question, many works have considered metabolic network modeling based on differential equations [3] and flux balance analysis (FBA) [4]. There have also been attempts to understand biological systems, including metabolism, from a network viewpoint—generally called *Network Biology* [5,6,7]. Metabolism can be defined as a series of chemical reactions, and is often presented as a network or graph, which consists of a set of nodes and edges (called *metabolic network*). Although simplification through such a network representation results in several gaps in biological information, these networks provide new insights into metabolism.

Thus far, many data analysis studies have discussed, in particular, the mechanisms involved in the formation (or evolution) of metabolic networks [8,9,10,11,12,13] and environmental adaptation from the viewpoint of metabolic networks [14,15]. It is believed that most positively selected mutations cause changes in metabolism, resulting in a better-adapted phenotypes based on natural history, phylogenetics, genetics, *etc* [16]; thus, metabolic networks are expected to be characterized by a long evolutionary history of adaptive shape-shifting with changing environments. For example, thermophiles possess metabolic enzymes with higher thermal stability. These thermo-stable proteins might be acquired through the selection of specific amino acid residues (e.g., charged residues) helping to increase protein structure stability. Due to changing nutrient availability and growth environments (e.g., pH and salt concentration), moreover, transporters and pumps might be newly obtained through horizontal gene transfer and/or its substrate specificity might be modified. As a result, the structure of metabolic network might be changed through the addition, deletion, and rewiring of metabolic pathways (see Section 3 and later for details).

Moreover, mathematical models for the formation and adaptation of metabolic networks have been constructed, based on current knowledge regarding metabolic networks. Despite the simplicity of these models, they have shown excellent agreement with empirical metabolic networks, and have helped in understanding the evolution and design principles of metabolic networks.

In recent years, in addition, these data analysis and theoretical approaches have been integrated with large-scale mutational analyses and laboratory evolution experiments, and this attempt provides deeper and more realistic understanding of evolution and environmental adaptation of metabolic systems at the molecular level (e.g., see [17,18]).

This review focuses on a wide range of data analysis and theoretical studies, including our previous studies, on metabolic systems from the network perspective. By reconsidering the findings obtained in these studies, we provide a bird’s-eye view of the formation and adaptation of metabolic networks. 

## 2. Representation of Metabolic Networks

Networks are represented as sets of nodes and edges drawn between the nodes; thus, networks are equivalent to graphs in mathematics. Network and graph are essentially synonymous and interchangeable in the field of graph theory. Networks are useful to represent and analyze metabolic systems because the systems are a series of chemical reactions. However, because of this simplicity of network representation, wide-ranging information on metabolic systems is ignored. For example, it is difficult to consider reaction stoichiometry, metabolic fluxes, cellular compartments, and so on when representing metabolic systems using networks. Thus, we need to be careful while representing (or modeling) metabolism as networks. To more comprehensively describe metabolic systems, the representation schemes based on XML (Extensible Markup Language) such as SBML (Systems Biology Markup Language) [19] and BioPAX (Biological Pathway Exchange) [20] are useful because these schemes can integrate different types of biological information using data structure. However, since the XML-based formats are complicated (*i.e*., they have many different types of information), we need to extract necessary information from these XML-based formats (*i.e*., the representation of metabolic systems needs to be simplified) for any purpose. Thus, we focus on metabolic networks as a simple model of metabolic systems although network representations have several limitations, explained as above. In this section, we introduce 2 major definitions of metabolic network representation and its limitations (see the following sections for details).

**Figure 1:**
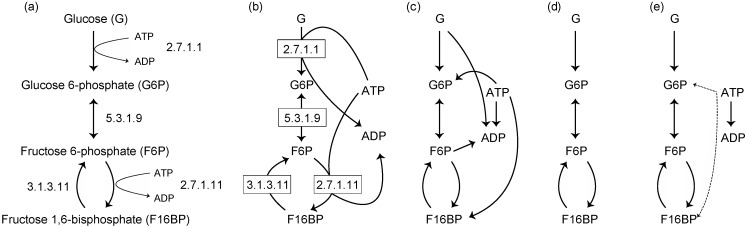
(**a**) A representation of a metabolic pathway as depicted in textbooks. (**b**) A representation using bipartite graphs. Examples of substrate–product networks based on chemical equations (**c**), based on chemical equations in which ubiquitous metabolites such as ATP and ADP are removed **(d)**, and based on atomic traces (**e**). The solid and dashed lines represent the traces based on carbon and phosphorus atoms, respectively.

Figure 1a shows a metabolic pathway as often depicted in textbooks. This network representation can be interpreted as a hypergraph because one edge can connect more than 2 nodes. On the other hand, metabolic pathways are also represented as a bipartite graph, which considers 2 types of nodes (compound nodes and enzyme nodes, in this case) (Figure 1b). For example, the pathway representation using bipartite graphs is used in databases on metabolism (e.g., KEGG). In these databases, metabolic systems are often represented using XML-based formats such as SBML and BioPAX. Although these representation schemes have high expression ability of metabolic systems, explained as above, network analysis methods and graph-theoretical algorithms for these network representations are relatively scarce.

**Figure 2:**
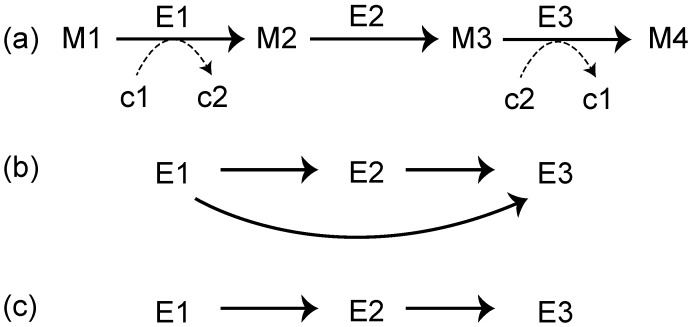
(**a**) A general representation of the metabolic pathway. Reactions are represented as enzymes (E1, E2, and E3). M1, M2, and M3 correspond to metabolic compounds. c1, c2, and c3 indicate currency metabolites such as ATP and NADH. The reaction network obtained from (a), without (**b**), and with (**c**) ubiquitous metabolites.

Consequently, metabolic network representations using simple graph and directed graphs, which are an unipartite graph, are well utilized (e.g., see Figure 1c–e and Figure 2b–c in which metabolic networks are represented as directed graphs). Since many of network analysis methods and graph-theoretical algorithms have been developed for such network representations (*i.e*. directed graphs and simple graphs), metabolic networks need to be represented using directed graphs or simple graphs in order to apply these existing methods and algorithms. In the following sections, 2 famous methods representing metabolic networks as directed graphs are introduced.

### 2.1. Substrate–Product Networks

Substrate–product network is a commonly used network representation, in which the nodes and edges are represented as chemical compounds (or metabolites) and substrate–product relationships in metabolic reactions, respectively. Note that this network representation is different from the representation using hypergraphs (Figure 1a). An edge is only drawn between 2 nodes (*i.e*., one edge cannot connect more than 2 nodes). As Arita [21] has pointed out, however, we need to be careful while defining substrate–product relationships. As a simple example, consider the following metabolic reaction: A + B →C + D. Based on the chemical reaction equation, the following substrate–product relationships may be obtained: A–B, A–C, B–C, and B–D [5,6] (see Figure 1c). However, some of these substrate–product relationships may not meaningful in terms of atomic traces. In particular, these problems arise when considering ubiquitous metabolites (also called currency metabolites) such as water and ATP.

For example, consider a metabolic reaction catalyzed by glucokinase (2.7.1.1): ATP + Glucose (G) →ADP + Glucose 6-phosphate (G6P). Even in this case, we obtain 4 possible substrate–product pairs. On the basis of the transfer of carbon atom groups, the substrate–product pairs obtained are ATP–ADP and G–G6P. The ATP–G6P pair is also obtained because of the transfer of a phosphate group. However, the G–ADP pair may be not acceptable in terms of atomic traces because of no atomic relationship between these metabolites. This problem also occurs in the bipartite graphs representation (Figure 1b): the G–ADP pair is obtained. 

Ubiquitous metabolites are connected to many other metabolites because of their ubiquity (*i.e*., such metabolites occur in many metabolic reactions); however, the connections with ubiquitous metabolites tend to include artifacts in terms of atomic traces (e.g., G–ADP pair in Figure 1c). These connections (edges) may disturb suitable network analysis. For example, when extracting metabolic pathways using algorithms finding *k* shortest paths in such networks, these extracted pathways may have no meaning in terms of atomic traces. 

To avoid this problem, ubiquitous metabolites are sometimes removed from metabolic networks (Figure 1d) [22,23]. However, since ubiquitous metabolites are acquired through metabolism, they should not be neglected (*i.e*., ubiquitous metabolites do not occur in metabolic networks, as shown in Figure 1d). In addition, definitions of ubiquitous metabolites may vary, and consequently pose an issue. Therefore, a method for identifying ubiquitous metabolites has been proposed using graph-clustering algorithms (e.g., see [23], in which the term *“currency metabolite”* is used as a synonymous of “*ubiquitous metabolite”*). 

Ideally, substrate–product relationships are defined by considering atomic traces (*i.e*., atomic position pairs between substrates and products corresponding to the substructural moieties conserved in each reaction) [21,24,25] (Figure 1e), as explained above in the example of a metabolic reaction catalyzed by glucokinase. This network representation can also consider substrate–product relationships between ubiquitous metabolites (e.g., ATP and ADP in Figure 1e). Note that these substrate–product relationships are ignored in Figure 1d. Moreover, substrate–product relationships can be distinguishable based on atomic types. In Figure 1e, for example, the atomic traces based on carbon atoms (solid arrows) and phosphorus atoms (dashed arrows) are shown. This consideration of atomic traces is complicated because the chemical structure of metabolites needs to be considered. However, reactant pairs based on atomic traces are available in several databases such as the RPAIR database [26] and Metabolomics.JP [27,28], in which the reactant papers among about 2000 types of metabolites are available; thus, it has been relatively easy to construct comprehensive metabolic networks based on atomic information. 

### 2.2. Reaction Networks

A reaction network is another popular representation of a metabolic network [22,29,30], and it is useful for analyzing the relationship enzymes and metabolic networks. The reaction network is obtained as follows. As an example, consider a metabolic pathway that consists of 3 metabolic enzymes: E1, E2, and E3 (Figure 2a). An edge is drawn between 2 reactions (nodes) if at least 1 product of a reaction corresponds to at least 1 substrate of the other reaction. For example, the link E1 →E2 is obtained because M2 acts as both the product of E1 and the substrate of E2. In this case, however, the ubiquitous metabolites, such as water, ATP, and NADH, generate links without essential biological roles. For example, we consider the reactions E1 and E3, whose interjacent chemical compound is the currency metabolite c2 (e.g., ATP). When considering metabolic reaction steps, it is not appropriate to draw an edge from E1 to E3 (Figure 2b) because ubiquitous metabolites play simple roles, such as energy exchange, exchange of a proton, or phosphate moiety; thus, the metabolic reaction network should be represented as in Figure 2c. To draw such networks, we need to distinguish ubiquitous metabolites (c1 and c2 in Figure 2) and non-ubiquitous metabolites (M1–M4 in Figure 2). In this case, the RPAIR database is useful. In this database, since substrate–product relationships are classified based on atomic traces; we can distinguish ubiquitous metabolites and non-ubiquitous metabolites.

## 3. Networks Provide an Extended View of Metabolic Evolution

To discuss metabolic networks, it is necessary to carefully consider the problems and limitations of the network representations. In particular, large-scale metabolic networks help to evaluate classical hypotheses in metabolic evolution. In this section, we introduce several data analysis studies on metabolic network evolution from a viewpoint of biomolecules such as enzymes and metabolites.

### 3.1. Roles of Gene Duplication

**Figure 3:**
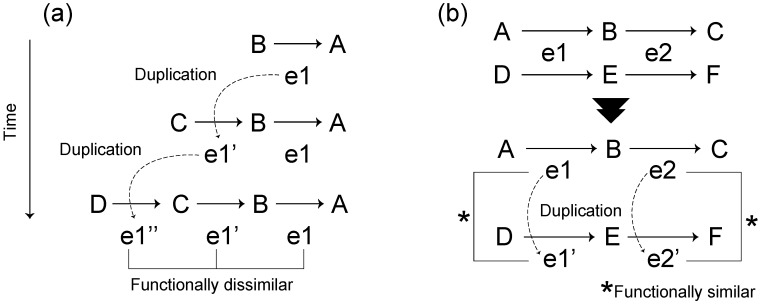
Schematic diagram of classical hypotheses in metabolic pathway evolution. (**a**) Retrograde evolution. Assuming that compound A is essential for survival and compound B is obtained from the external environment, survival is dependent on the existence of compound B in the environment. When compound B decreases due to perturbations, a new enzyme e1′ that can synthesize the compound B from compound C, which is more widely available in the environment, is acquired through gene duplication of the enzyme e1. (**b**) Patchwork evolution considers that the present metabolic pathway is obtained by substrate-specific specialization of ancestral enzymes with broad substrate specificities via gene duplication. Due to broad substrate specificities, the enzyme e1 (e2) catalyze both reactions between metabolites A and B (B and C) and between metabolites D and E (E and F) (the upper pathway). Though the duplication of e1 (e2), the enzyme e1’ (e2’) emerges. Due to substrate-specific specialization, the enzymes e1 (e2) and e1’ (e2’) catalyze the reaction between metabolites A and B (B and C) and the reaction between metabolites D and E (E and F), respectively (the lower pathway).

The evolution of metabolic pathways has been discussed since the mid-twentieth century, and several hypotheses have been proposed. In general, these hypotheses assume the expansion of a metabolic pathway based on evolutionary events such as gene duplication and horizontal gene transfer. There are 2 famous hypotheses for metabolic pathway evolution:

The first hypothesis is that of retrograde evolution proposed by Horowitz in 1945 [31]. This evolutionary hypothesis states that metabolic pathways assembled backwards, compared to the direction of the pathway, in response to depletion of substrates from the environment through gene duplication (Figure 3a). This evolutionary mechanism predicts the formation of dissimilar reactions within the same pathway or a neighboring part of the metabolic network [30,32]. 

The second hypothesis is patchwork evolution [33] (often referred to as the recruitment evolution [34]), which states that enzymes initially have broad substrate specificities and that specialization occurs by means of gene duplication (Figure 3b); thus, this evolutionary mechanism predicts that duplicate pairs of genes are functionally similar.

These hypotheses were partially validated in specific metabolic pathways. For example, the consecutive steps of tryptophan biosynthesis [35] and the fact that the TIM-barrel-containing enzymes have been found in many different pathways [36] support the retrograde evolution and patchwork evolution hypotheses, respectively.

Which hypothesis is more suitable? Thus far, there is no definite answer to this question due to limited evaluations (*i.e*., evaluation of specific metabolic pathways). In recent years, however, these hypotheses have been evaluated through large-scale metabolic network analyses, and as a result, considerable progress has been made.

Zhang *et al.* [32] showed that enzymes sharing the same folds have similar enzymatic functions in a thermophilic bacterium, and there are few such enzymes in the vicinity of each other in a metabolic network. This finding provides an example of patchwork evolution. However, they also concluded that pathway evolution through retrograde evolution could not be neglected because the probability that structurally similar enzymes are contiguous in metabolic pathways is still larger than that expected by change. 

**Figure 4:**
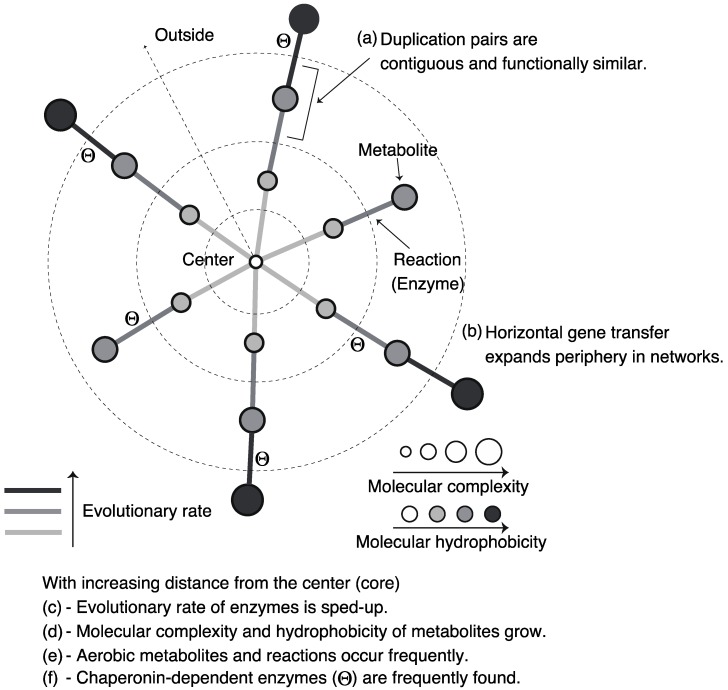
Schematic diagram of the roles and properties of enzymes and metabolites in metabolic networks.

Díaz-Mejía *et al.* [29] and Light and Kraulis [30] investigated the contiguity of duplication pairs in the metabolic networks of *Escherichia col*, represented as reaction networks, using the shortest path length (defined as the number of edges in the shortest path between a node pair). In reaction networks, shortest path length indicates the minimum number of reaction steps. They found that the shortest path length between homologous pairs is smaller than that expected by chance. This result suggests that duplication pairs are neighboring in the metabolic networks, as predicted by retrograde evolution. However, the homologous pairs showed similar enzymatic functions, suggesting patchwork evolution. Since the proximity of duplication pairs with dissimilar functions in metabolic networks is more significant than that of duplication pairs with similar functions, Light and Kraulis concluded that retrograde evolution plays a limited role in metabolic pathway expansion, and that patchwork evolution is the predominant mechanism underlying metabolic pathway expansion. Díaz-Mejía *et al*., however, suggested that retrograde evolution and patchwork evolution might not be independent of each other because the proximity of duplication pairs is statistically significant despite functional similarity (Figure 4a). For example, the origin of 4 homologous carbon–nitrogen ligases catalyzing consecutive reactions in peptidoglycan biosynthesis is consistent with both retrograde and patchwork evolution [30].

### 3.2. Role of Horizontal Gene Transfer

In the previous section, we only mentioned the roles of gene duplication in metabolic pathway evolution. In eukaryotes, gene duplication may be the main driving force of metabolic evolution; thus, the explanation of pathway evolution based on gene duplication is suitable. In bacteria, however, it is believed that metabolic pathway expansion is caused by horizontal gene transfer, which is the alternative driving force of evolution, rather than gene duplication, because a comparison between *E. coli* and *Saccharomyces cerevisiae* (yeast) showed that bacteria have few duplicated enzymes in metabolic networks. 

Pál *et al.* [37] suggested that horizontal gene transfer plays a role in metabolic evolution, and their findings showed a correlation between enzymatic functional classes and frequencies of horizontal gene transfer in its classes in the *E. coli* metabolic network. In particular, they showed that metabolic genes acquired through horizontal gene transfer are highly observed in transporter-related enzymes although such genes are hardly found in central-metabolism-related enzymes, suggesting that the periphery of bacterial metabolic networks evolve through horizontal gene transfer (Figure 4b). Transporter-related enzymes are important for the response to changing environments (e.g., nutrient availability). Thus, bacterial metabolic networks might adaptively shape-shift with the changing environment through horizontal gene transfer.

### 3.3. Evolutionary Rate in Metabolic Networks

The previous sections considered the gain of metabolic enzymes through gene duplication and horizontal gene transfer. Also, genes constantly undergo mutations, and sometimes, these mutations may result in metabolic evolution. It is natural to question whether a relationship exists between the position of an enzyme (e.g., distance of an enzyme node from the center) in metabolic networks (represented as reaction networks) and the evolutionary rate of the enzyme, defined as the ratio of the non-synonymous substitution rate to the synonymous substitution rate in this review.

Indeed, a correlation between the position of an enzyme in metabolic networks and the evolutionary rate of the enzyme was observed: the evolutionary rate of centric enzymes is lower than that of peripheral enzymes in metabolic networks (Figure 4c). This is called *evolutionary rate variation*. This tendency implies the rapid evolution of peripheral metabolic networks, and may be related to peripheral evolution of metabolic networks, explained in Section 3.2. The central metabolic networks are essential for survival; thus, the evolutionary rate of centric enzymes may be low.

Historically, this tendency was found in the biosynthesis pathway of anthocyanin, a kind of secondary metabolite in a plant (morning glory) [38]. Here, the degree of centrality of enzymes in the metabolic pathway is indicated by the number of reaction steps from the source enzymes. Ramsay *et al.* [39] investigated the terpenoid biosynthesis pathway, and showed that the evolutionary rate variation is appreciable in wider secondary metabolic pathways. Moreover, Vitkup *et al.* [40] found a negative correlation between the evolutionary rate and degree of centrality of enzymes, which can be calculated using graph-theoretic centrality measures [41], in the large-scale metabolic network (reaction network) of yeast, and they suggested that the evolutionary rate variation is a general tendency in metabolic networks.

### 3.4. From a Viewpoint of Chemical Properties of Metabolites

When discussing metabolic evolution, chemical properties of metabolites are also important in addition to metabolic enzymes.

Through a data analysis and theoretical model, Holme [42] showed a negative correlation between molecular mass (*i.e*., the number of atoms in a metabolite) and the node degree in metabolic networks of humans, represented as substrate–product networks. This finding suggests that more structurally simpler metabolites are converted to complex metabolites through metabolic reactions (Figure 4d).

This tendency might be explained by the Granick hypothesis [8,10], which proposes a forward evolution in which biosynthetic pathways develop via the formation of more complex molecules from simple molecules. In particular, this hypothesis predicts a negative correlation between molecular complexity and node degrees in substrate–product networks because high-degree metabolites (*i.e*., sources of other metabolites) are expected to be primitive.

Using chemoinformatics, Zhu *et al.* [43] also investigated the chemical basis of metabolic networks of *E. coli* and yeast in more detail, and they showed that high-degree metabolites have strong polarity or hydrophilicity (Figure 4d). Since life might have originated from aquatic environments, the primordial metabolites may be highly hydrophilic. In addition to this, the hydrophobicity is related to metabolite concentrations, suggesting that the chemical properties of metabolites influence the evolution of metabolic systems [44]. Moreover, further investigation [45] by chemoinformatics revealed the effect of oxygen on metabolic evolution by comparing chemical properties of metabolites between aerobic and anaerobic reactions. In particular, it was found that aerobic metabolism involves diverse chemical structures. This result is consistent with the expansion of metabolic networks due to oxygen availability [46]. Furthermore, many aerobic metabolites are related to environmental adaptations such as defense against biotic factors and protection of organisms against oxidation. In addition, aerobic metabolites seem to be hydrophobic and rigid (Figure 4e). This tendency is consistent with the fact that membranes function to serve such environmental adaptations.

### 3.5. Roles of Chaperonin

Protein folding is an important aspect of metabolism because normal metabolism requires proper functioning of cellular enzymes (*i.e*., the satisfactory conformation and function of native enzyme structures). Chaperones, most of which are heat-shock proteins, assist in protein folding, and they prevent misfolding and aggregation of proteins (reviewed in [47,48]). In particular, in *E. coli*, the chaperonin GroEL, together with its cofactor GroES, acts as a chaperone system.

Previous comprehensive analyses [49,50] identified substrate proteins for which folding requires the GroEL in *E. coli*, and they revealed that about 70% of substrate proteins are metabolic enzymes. This result shows the importance of GroEL in metabolism. However, the relationship between GroEL and metabolism is still unclear.

Takemoto *et al*. [51] found that as GroEL requirement increases, substrate enzymes are distributed on the periphery of the metabolic network (Figure 4f). In addition, comparative genome analysis showed that the GroEL-dependent substrates were acquired later on in evolutionary history. This result implies the expansion of metabolic networks due to GroEL.

Historically, it is believed that chaperones, including chaperonin, are related to evolution. Several works [52,53] have shown that a chaperone (Hsp90 in this case) can promote genetic and phenotypic diversity (*i.e*., morphological evolution) because it functions as a buffering systems genetic mutation by helping protein folding. Inspired by this study, Tokuriki and Tawfik [54] reported the modification of enzymatic specificity (*i.e*., change in enzymatic function) induced by the overexpression of GroEL through experimental evolution. Moreover, it has been shown that the GroEL accelerates the evolutionary rate of proteins in *E. coli* [55,56], and this result is consistent with the evolutionary rate variation. For these reasons, the increase in enzymatic diversity due to GroEL is expected to cause expansion of metabolic networks.

The expansion of metabolic networks through GroEL provides new insights into metabolic evolution and the roles of chaperonins in living systems. Other chaperones such as Hsp90 are also expected to play roles in metabolic network evolution because phenotypes are related to metabolism.

## 4. Understanding Formation and Adaptation through Structural Properties

The topology of metabolic networks, characterized by graph-theoretic techniques, also exemplifies the formation and adaptation of metabolism. Especially, several striking structural properties have been found in metabolic networks, and their relationship with environments has been actively investigated. However, since network representations influence these structural properties, these properties need to be interpreted carefully. Despite the problems and limitations of metabolic network representations, appropriate discussions are possible when carefully considering the problems and limitations. In this section, we introduce several data analysis studies, which discuss metabolic network evolution and adaptation through structural properties. 

### 4.1. Scele-Free Connectivity

**Figure 5:**
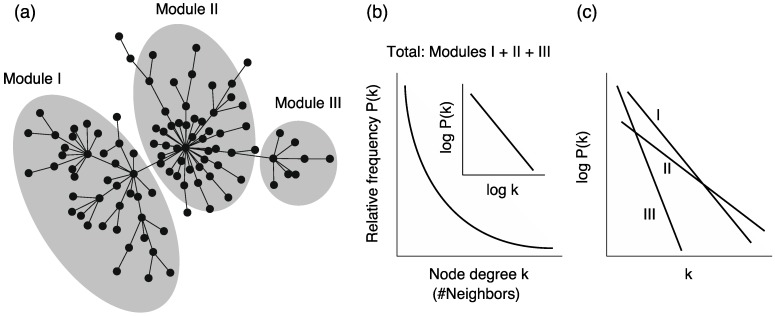
**(a)** Schematic diagram of heterogeneous connectivity in metabolic networks. **(b)** The node degrees in whole networks follow a power-law distribution. Note that the inset graph is a double logarithmic plot. **(c)** On the other hand, the node degree in each module (sub-network) follows an exponential distribution, and the distributions are different between the modules. Note the single logarithmic plot.

*Scale-free connectivity (or heterogeneous connectivity)* is a well-known structural property; it implies that a few nodes (hubs) are connected to numerous nodes, whereas the remaining nodes are not (Figure 5a). In particular, it is empirically known that the node degree *k* (the number of edges that a node has) follows a power-law-like distribution [57,58,59]: 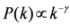, where *P(k)* is the relative frequency of nodes with degree *k* (Figure 5b).

Hubs may play important roles in metabolic networks. In reaction networks, for example, hub enzymes show low evolutionary rates, suggesting that they are strongly constrained in terms of evolution [40]. In substrate–product networks, hub metabolites have high concentrations in cells [43], indicating that they are key players that govern metabolic dynamics. However, hub metabolites vary among the different definitions of metabolic network representation (see Table 1 in [21]); although power-law-like distributions are observed, the exponent *γ* varies among the definitions. Therefore, care needs to be taken when interpreting results obtained from metabolic networks.

In such networks, the average degree is not representative although it can be calculated in networks of a finite size. Networks that have this statistical property are called *scale-free networks*.

The scale-free property (power-law distribution) is often used in the sense of scale invariance (*i.e*., self-similarity) because 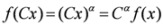 is satisfied, where *C* and *α* are constants, when considering a power-law function 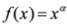.

In fact, Song *et al.* [60], using the extended box-counting method, which calculates the fractal dimension of a self-similar material, showed that empirical metabolic networks have self-similarity.

However, the relationship between self-similarity and scale-free networks has sometimes been criticized [61]. For example, we can generate a self-dissimilar network whose degree distribution follows the power law [62]. Moreover, metabolic networks are suggested to be self-dissimilar because the degree distributions among different biosynthetic modules are very different [63] (Figure 5c). In particular, it was found that the power­-law distribution in the whole network is obtained from the summation of the exponential distributions in reaction modules.

This self-dissimilar property is called *scale-richness*, as opposed to scale-freeness. For the above reasons, we need to discuss the relationship between power-law distributions and self-invariance (self-similarity) carefully in metabolic networks.

### 4.2. Small Worldness

**Figure 6:**
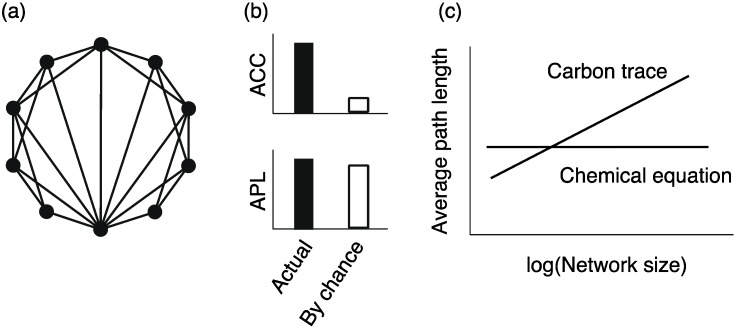
**(a)** Schematic diagram of small-world metabolic networks. **(b) **In general, the small-world properties are defined according to the statistical significance of the average clustering coefficient (ACC) and average path length (APL). **(c)** The network size dependency of APL is different between network representations.

Interestingly, in networks, the distance between a given node pair is surprisingly small although the network size is very large and locally clustered (Figure 6a). This property is referred to as the *small-world property* and was originally known as the *6 degrees of separation* in sociology [64].

The distance between a node pair can be measured using the average shortest path length of a network, which is defined as the shortest path length averaged over all pairs of nodes, and the degree of local clustering of a network is measured using the clustering coefficient averaged over all nodes. The clustering coefficient of node *i* is defined as 
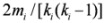
, where *m_i_* and *k_i_* indicate the number of edges among neighbors of node *i* and the degree of node *i*, respectively.

The small-world property is also observed in metabolic networks [22]. The clustering coefficient is larger than that obtained by chance, and the average path length is almost equivalent to that acquired by chance (Figure 6b).

However, the average path length and average clustering coefficient vary according to network representations [21]. The average path length is particularly significant. The average path length calculated from substrate–product network representations based on atomic traces is 2.6 times larger than that based on chemical reaction equations. This finding sheds doubt on the small-world property of metabolic networks. Furthermore, while the average path length calculated from substrate–product networks based on chemical reaction equations is independent of the network size [5] (*i.e*., the number of metabolites), the average path length of substrate–product networks based on carbon traces increases logarithmically with the network size [24] (Figure 6c).

### 4.3. Preferential Attachment Mechanism

**Figure 7:**
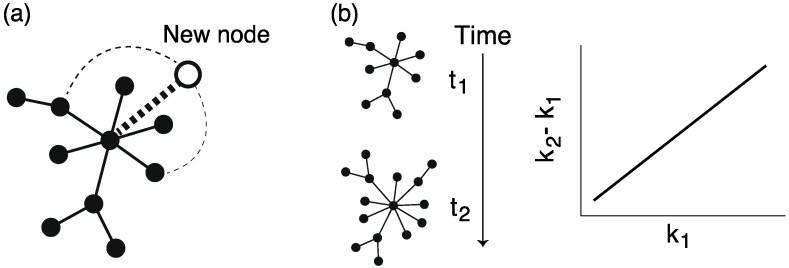
**(a)** Schematic diagram of the preferential attachment mechanisms. The dashed lines correspond to possible links. The width of the dashed lines indicates the probability that the possible links are selected. Note that only 3 possible links are shown in this figure although possible links exist between the new node (white node) and all existing nodes (black nodes). **(b) **The preferential attachment can be evaluated according to the difference in node degrees between the networks at different times: *k_2_ – k_1_*, where *k_1_* and *k_2_* are the node degree in the networks at times *t_1_* and *t_2_*, respectively.

What is the origin of heterogeneous connectivity in metabolic networks? Barabási and Albert [7,57] showed that the heterogeneous connectivity is acquired by 2 mechanisms: the growth mechanism, in which an added node connects to existing nodes, and the preferential attachment (or rich-get-richer) mechanism, in which an existing node obtains new edges based on the probability that is proportional to its node degree (Figure 7a). Do metabolic networks have these mechanisms?

Light *et al.* [65] validated the preferential attachment mechanism in metabolic networks represented as reaction networks. They classified enzymes into 5 phylogenetic groups according to their conservation degree, and investigated the changes in node degrees across the phylogenetic groups. Their study revealed that the change in node degree of higher-degree nodes is larger than that of lower-degree nodes (Figure 7b), suggesting the preferential mechanism. In general, gene duplication is derived from the preferential attachment mechanism [29,66,67,68,69]. However, the preferential attachment mechanism is observed not only in enzymes acquired through gene duplication but also in those obtained through horizontal gene transfer, suggesting that this mechanism is fundamental in metabolic network evolution.

Furthermore, a theoretical study [70] based on artificial enzymatic reactions discussed the emergence of heterogeneous connectivity in metabolic networks, and showed that a number of highly specialized enzymes might evolve from a few multifunctional enzymes through gene duplication, as predicted by patchwork evolution, and that group transfer reactions are essential for the emergence of hubs.

In addition, the preferential attachment mechanism based on metabolite concentration has also been proposed [43]. According to this, metabolites that are present in abundant quantities drive a variety of reactions. The empirical metabolite concentrations slightly correlate with node degrees, suggesting the role of metabolite concentration in metabolic network formation.

### 4.4. Network Modularity

It is believed that biological systems (networks), including metabolism, are modularized [71]. In fact, metabolic networks consist of linked functional components or modules. This structural tendency can be characterized by network modularity, which, in essence, reflects the deconstruction of a network into dense, and yet, weakly interconnected sub-networks [72,73] (Figure 7). Although the definition of network modularity is an open question, a network modularity measure, called *Q-value* (e.g., see Equation 1 in [73]), has provided insight into metabolic network formation and adaptation.

**Figure 8:**
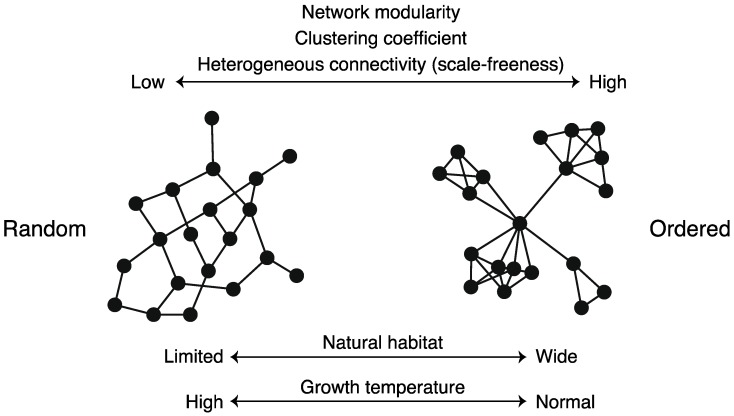
Schematic diagram of structural properties of metabolic networks. Network structure is related to species’ natural habitats, growth temperature, *etc*.

For example, Parter *et al.* [74] showed that variability in a natural habitat promotes metabolic network modularity in bacteria (*i.e*., the network modularity of an organism living in wider environments is higher) (Figure 8). This study was inspired by a theoretical study [75] that states that modular networks spontaneously evolve when the evolutionary goal (*i.e*., system-specific purpose) changes over time in a manner that preserves the same sub-goals but in different permutations.

However, further data analysis and investigations have revealed an exception. Takemoto and Borjigin [76] found the absence of a relationship between network modularity and habitat variability in archaea, as archaeal habitats are more limited than bacterial habitats. Further investigations revealed alternative explanations. In particular, growth conditions, trophic requirement, and optimal growth temperature, decrease metabolic network modularity (Figure 8). It has been reported that the growth temperature also diminishes the clustering coefficient, related to network modularity, of metabolic networks [77]. Especially, metabolic networks undergo a change from heterogeneous and high-modular (*i.e.*, ordered) structures to homogeneous and low-modular (*i.e.*, random) structures, such as random networks, with temperature [77] (Figure 8). This finding suggests that the temperature plays an important role in the design principles of metabolic networks.

Parter *et al.* [74] explained that network modularity decreases as alternative paths between a given metabolite pair disappear in organisms whose habitats are narrow. This disappearance of alternative paths indicates that network modules are broken (*i.e*., decay of network modularity occurs). However, another work [78] uses the network model to show that such an alternative path disappears (*i.e*., is not selected) at a high optimal growth temperature. The selection of alternative paths might be caused by a temperature-dependent selective constraint (negative selection) [79,80]. Metabolic pathways consist of enzymes (*i.e*., proteins). Because enzymes might need structural stability to survive, in hot environments, they tend to get easily deactivated, and therefore, the emergence of alternative paths may be restricted.

## 5. Reconstruction of Ancestral Metabolic Networks

Large-scale data on metabolic networks are available in several databases; however, data regarding ancestral metabolic networks are unavailable. Therefore, there have been attempts to reconstruct (estimate) ancestral metabolic networks using observed (current) metabolic information (*i.e*., gene contents) and phylogenetic profiles (mainly, phylogenetic trees). Reconstruction of ancestral networks is a powerful tool for understanding metabolic network formation by tracing it. These estimation methods are divided into 2 major types.

The first is an estimation method based on the principle of maximum parsimony [81], in which ancestral networks are estimated by minimizing the number of evolutionary events, such as a gain/loss of enzymes, according to the topology of a phylogenetic tree. Because of the simplicity of this method, it is widely used to reconstruct ancestral networks; however, this method hardly considers that heterogeneous evolution, e.g., genome duplication and parasitization, respectively, result in high rates of gene gain and loss.

The other estimation method is based on maximum likelihood [82,83] that considers a stochastic process in which gain/loss of gene occurs with a general probability. By selecting the best evolutionary scenario (model) reproducing the present gene contents (*i.e.*, by maximizing a likelihood), ancestral networks are reconstructed. Maximum likelihood-based methods assume different rates of gene gain/loss at each evolutionary step, and thus consider heterogeneous evolution; therefore, maximum likelihood-based methods are better than maximum parsimony-based methods.

These methods of reconstructing ancestral networks have revealed metabolic network formation in more detail. For example, Iwasaki and Takagi [84] showed that metabolic networks might be discretely expanded through massive horizontal gene transfers as gene groups. This finding is strongly related to the punctuated equilibrium theory [85] in evolution.

Furthermore, Kreimer *et al.* [86] found that the decrease in network modularity is due to niche specialization and the addition of peripheral metabolic pathways occurs during evolution. Caetano-Anollés *et al.* [87] discovered that the most ancestral metabolic network consisted of nucleotide metabolism, and they concluded that this result supports the RNA-world hypothesis for the origin of life.

Tanaka *et al.* [88] found that the preferential attachment mechanism is observed in prokaryotic metabolic networks, but not in eukaryotic metabolic networks. This different evolutionary pattern suggests that, in bacteria, as opposed to eukaryotes, gain and loss of enzymatic reactions have direct effects on key metabolites such as L-aspartate and acetyl-CoA.

**Figure 9:**
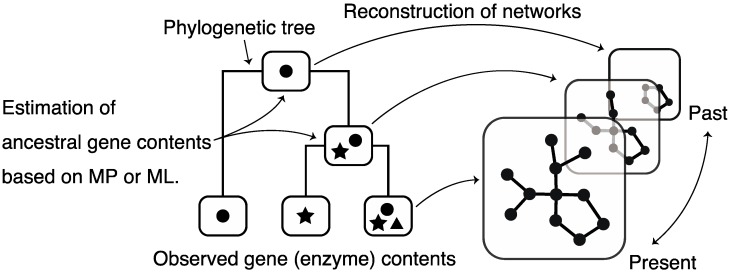
Schematic diagram of the reconstruction of ancestral metabolic networks. MP: maximum parsimony, ML: maximum likelihood.

## 6. Measuring Metabolic Network Robustness

Robustness is a key feature in biological systems [89], including metabolism, because many organisms have strong adaptability to environmental changes or failures in some of their components, and they can live even if some of their genes are mutated.

Ideally, metabolic dynamics such as changes in enzyme expression levels and metabolite concentrations should be considered when discussing metabolic robustness. For this, kinetic parameters are required in general; however, such data are largely unknown. 

Therefore, many studies have focused on the structural robustness of metabolic networks, which refers to the tolerance of the system’s behavior to changes in the structure of metabolic networks, and these studies consider changes caused by knockout of genes or enzymes. Kinetic parameters are not required for the evaluation of structural robustness. Structural robustness can be evaluated by 2 major methods:

The first is the method based on constraint models [4] such as the flux balance analysis (FBA) and elementary flux modes (EFMs)—an EFM is the minimum set of reactions that can occur at a steady state. For example, Burgard *et al.* [90] focused on finding a minimum reaction cut based on the FBA, that is, a minimum set of reactions (or enzymes) whose removal prevents the production of a specified set of compounds (*i.e.*, biomass formation). In another approach, Behre *et al.* [91] proposed a measure based on the number of remaining EFMs after knockout versus the number of EFMs in the unperturbed situation. In this manner, the FBA and EMFs provides different insights into metabolic robustness, respectively. These methods are also useful for deeper understanding of metabolic pathway evolution because metabolic systems might evolve to increase their robustness. 

The other method is based on Boolean models of metabolic networks, in which reactions and compounds are modeled as AND and OR nodes, respectively. Handorf *et al.* [92] analyzed robustness of metabolic networks by introducing the concept of scope. Furthermore, Smart *et al.* [93] defined the topological flux balance (TFB) criterion based on a Boolean model of metabolic networks and analyzed the damage (number of reactions) caused by knockout of a single reaction under TFB. Jiang *et al*. [94] defined and analyzed the impact degree, which is the number of reactions inactivated by knockout of a specific reaction. Although there are some differences in the treatment of reversible reactions, damage and impact degree are very similar concepts. Moreover, the TFB (or impact degree) can be analytically estimated using the branching process [95], which is a stochastic process in which each progenitor generates offspring according to a fixed probability distribution called the offspring distribution.

## 7. Mathematical Models for Metabolic Network Formation

Mathematical models for the formation and adaptation of metabolic networks have been constructed according to the current understanding of metabolic networks from a network perspective. Despite the simplicity of these models, they have shown excellent agreement with empirical metabolic networks, and they are helpful for understanding the evolution and design principles in metabolic networks. Similarly, the theoretical construction of models for network formation has also been attempted in other biological networks such as protein–protein interaction networks [66] and gene regulatory networks [69].

The toolbox model [96] is a well-known model for metabolic networks, represented as substrate–product networks, and it assumes that the metabolic network of a given organism constitutes a subset of the universal biochemistry network, formed by the union of all the metabolites and metabolic reactions taking place in any organism. In particular, the metabolic network of an organism arises from self-avoiding random walks on the universal network. This model is in agreement with empirical metabolic networks. In particular, this model shows a relationship with transcriptional regulation, and it can explain the quadratic scaling of the number of transcription factors, which is an empirical statistical law. Since adaptation to a new environmental condition is monitored by a new transcription factor (e.g., learning to use another nutrient), the toolbox model can help to understand adaptation mechanisms from a transcriptional regulatory and metabolic perspective. However, this model is restricted to prokaryotic catabolic pathways and requires universal biochemistry networks even though model networks are only slightly influenced by the topology of the universal network [97].

Takemoto and Akutsu [78] proposed a more general model for the formation of metabolic networks, represented as substrate–product networks. Although this model does not consider the relationships inherent in transcriptional regulation explained by the toolbox model, it is applicable to metabolic networks, including both catabolic and anabolic pathways, and to prokaryotes and eukaryotes. Moreover, parameter tuning is not necessary although this model requires several parameters; we can readily estimate the model parameters by using the simple graph-theoretic statistics of an empirical metabolic network such as the number of nodes, number of edges, *etc*. Despite its simplicity, this model is in excellent agreement with empirical data, and it has explained the origin of structural differences in metabolic networks with respect to growth temperature [76,78]. This model is also expected to explain environmental adaptation.

## 8. Metabolite Distribution across Species

In addition to considering metabolic networks, however, it is also important to consider how metabolites are distributed among species in order to elucidate design principles of metabolism such as adaptive mechanisms. Metabolite distribution has the following advantages. Since living organisms have specific metabolite compositions due to metabolisms adaptively changing with respect to the environment, we can estimate environmental adaptation (adaptive evolution) using metabolite distributions (*i.e.*, species–metabolite relationships). Moreover, they are also useful for characterizing species relationships, which are highly linked to ecological systems. For metabolite distribution, thus, identification of structures and construction of a theory (model) for evolutionary mechanisms are key challenges for a deeper understanding of metabolism.

The relationship between species and metabolites can be represented as a *bipartite network*. Bipartite networks are defined as graphs having 2 different node sets (in this case, species and metabolites), in which edges are only drawn between 1 node set and the other node set (interconnectivity). Notably, there is no edge between the nodes belonging to the same node set (intraconnectivity). In the species–metabolite network, an edge is drawn between a species node and a metabolite node when the species has the metabolite.

**Figure 10:**
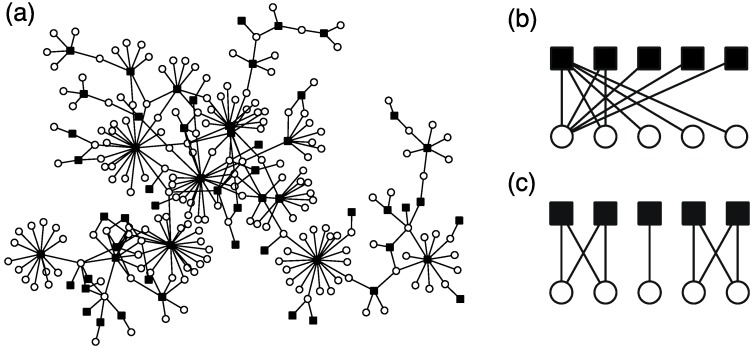
**(a)** The heterogeneous metabolite distribution (species–metabolite network) of Rutaceae (citrus family). Schematic diagram of the reconstruction of nested structure (b) and modular structure (c). The black squares and white circles correspond to species and metabolites, respectively.

In particular, the metabolite distributions of flavonoids, a type of secondary metabolite in plant species, are well investigated because of the great divergence of flavonoids. By analyzing this bipartite network, the following universal properties of flavonoid distribution were revealed despite different compositions of flavonoids among families [98,99]. (i) The number of flavonoids in a species and the number of plant species sharing a flavonoid follow a heterogeneous (power-law-like) distribution (Figure 10a). That is, most metabolites are shared by a few species; however, a few metabolites are conserved in many species. Most species have metabolites of a few types; however, a few species have metabolites of many types. This property is related to the scale-free property. (ii) A plant’s metabolite composition is a subset of another plants’ metabolite composition. (*i.e.*, nested structure) (Figure 10b). (iii) Plant species are divided into several clusters based on the composition of their metabolites (*i.e.*, modular structure) (Figure 10c).

Moreover, a possible origin of these structural properties was proposed [98,99], and these striking properties are reproduced through simple evolutionary processes: the emergence of new species due to mutations and the development of new flavonoids through modifications in the existing flavonoids.

## 9. Metabolism in Ecosystems

Up until now, many studies have focused on metabolic networks of individual species. However, it will become more important to consider metabolic networks in ecosystems. In particular, species–species interactions are interesting when discussing the formation and adaptation of metabolic networks. As explained in Section 3.2, the horizontal gene transfers expand metabolic networks, suggesting that species–species interactions influence metabolic networks. Bridging the gap between individual metabolic networks and larger networks of species communities is highly relevant not only to metabolic engineering but also to biomedical science and earth-system science [100]. In particular, since metagenomic sequencing data provide a snapshot of the microbial communities genetic contest, the relationship between individual metabolic networks and ecosystems is progressively becoming clearer.

For example, Christian *et al*. [101] and Borenstein and Feldman [102] used the concept of scopes [92] and, which describe biosynthetic capabilities, and seed sets [103], which correspond to exogenously-acquired metabolites, and they discussed a relationship between species–species interactions (symbiotic and parasitic interactions) and metabolic networks, respectively. Especially, Christian *et al*. showed that the metabolic capability increases due to mutual cooperation, and Borenstein and Feldman revealed that the biosynthetic capability decayed in the *Firmicutes* division due to mutual cooperation in evolutionary history, by reconstructing ancestral metabolic networks.

FBA-based metabolic models also contribute to understand metabolism in ecosystems [4]. Especially, Freilich *et al.* [104] predicted the competitive and cooperative potential between bacterial pairs obtained from a collection of about 100 species’ FBA-based metabolic models, and they comfirmed the high level of competition and unidirectional cooperative interactions among species using ecological data from about 3000 samples.

## 10. Large-Scale Mutational Analyses and Laboratory Evolution Experiments

The findings from the above data analyses and theoretical approaches are still hypothetical. For deeper and realistic understanding of formation and adaptation of metabolic systems, experimental approaches are necessary; thus, large-scale mutational analysis and laboratory evolution experiments have actively performed with theoretical approaches (constraint-based models such as FBA and EMFs in particular) in recent years [17,18].

For example, Ibarra *et al*. [105] found that *E. coli* adaptively evolved to show optimal growth rates expected from a constraint-based model although constraint-based models sometimes fail in predictions of growth rates. This result suggests that incomplete adaptive evolution causes failure in predictions of growth rate using constraint-based model. Similarly, Fong and Palsson [106] showed that a constraint-based model could predict the growth rates of gene-deletion strains of *E.coli* in most cases, and they found that gene-deletion strains evolved to increase their growth rates (*i.e.*, adaptive evolution) and that the strains evolved in parallel under identical nutrient conditions. Moreover, Lewis *et al.* [107] confirmed that transcriptomic and proteomic data also support activate reactions expected from constraint-based models in the wild-type and evolved strains. 

These findings imply that metabolic systems evolved to increase fitness (e.g., growth rates) with changing environments. Thus, it can be expected that the metabolic networks also changes with changing environments, as predicted from the data analyses and theoretical models in the previous sections.

The above studies mainly focus on nutrient conditions as environments. On the other hand, large-scale mutational analyses and laboratory evolution experiments have been also perform to reveal adaptive evolutions that achieve a high-temperature tolerance [108] and ethanol tolerance [109]. Although the interposition of theoretical models into these adaptive evolutions are still poorly, the integration of experiments and theories promote a deeper understand of formation and adaptation of metabolic systems.

## 11. Conclusions

This review has described the current understanding of metabolic network formation and adaptation revealed through data analysis and theoretical studies. Specifically, the classical view of metabolic evolution such as retrograde evolution and patchwork evolution has been largely confirmed, and several novel findings regarding formation and adaption, such as the role of chemical properties of metabolites and chaperonin in metabolic networks and the relationship between metabolic network structure and environmental factors, have also been discussed. Moreover, recent mathematical models for metabolic network formation have also described. 

The current understanding and mathematical models are still limited because they remain hypothetical; thus, further evaluations and biological evidences are required in order to complete the understanding of metabolic network formation and adaptation. For this, we need to consider the construction of more accurate databases and further development of effective analytical tools of metabolic networks in the future. In addition to, the large-scale mutational analyses and laboratory evolution experiments and are also useful for hypothesis testing and deeper understating of evolution and adaptation of metabolic systems. 

Nevertheless, these findings and mathematical models indicate global mechanisms for metabolic evolution and adaptation, and they are helpful in the field of not only general biology but also biotechnology, e.g., metabolic engineering. Furthermore, knowledge of evolutionary selection and environmental adaptation of enzymes in metabolic networks is directly linked to the modification and design of metabolic systems, and it is expected to establish a basis for identifying enzymes and metabolites with a great deal of potential in industry. Moreover, the mathematical models may be used to devise simple methods for determining important metabolic reactions and predicting interactions between biomolecules (*i.e.*, link prediction, described in [110]). For example, the TFB or impact degree is useful for easily identifying key metabolic reactions. Enzyme promiscuity [111], which implies that enzymes can catalyze multiple reactions, act on more than 1 substrate, or exert a range of suppressions [112], in which an enzymatic function is suppressed by overexpressing enzymes showing originally different functions, suggests the existence of many hidden metabolic reactions. These biological features in metabolism are important for designing metabolic pathways and understanding metabolic evolution [113,114]; the models may be helpful in identifying such hidden metabolic reactions.

In addition to this, the analysis and modeling of metabolic networks in ecosystem will become more exciting. Especially, they may be useful for understanding and controlling enteric, soil, and marine environments thorough microbes by combining metagenomic data [115,116,117]; thus, studies on metabolic networks in ecosystem will be more important in terms of medial ecological sciences in addition to evolutionary biology and metabolic engineering.

Considering that metabolic networks have not been fully understood, network biology presents a research field with high future growth potential.
